# Targeted metabolomics of CSF in healthy individuals and patients with secondary progressive multiple sclerosis using high-resolution mass spectrometry

**DOI:** 10.1007/s11306-020-1648-5

**Published:** 2020-02-12

**Authors:** Henrik Carlsson, Sandy Abujrais, Stephanie Herman, Payam Emami Khoonsari, Torbjörn Åkerfeldt, Anders Svenningsson, Joachim Burman, Kim Kultima

**Affiliations:** 1 Department of Medical Sciences, Clinical Chemistry, Uppsala University, Uppsala University Hospital, Entrance 61, 3rd Floor, Dag Hammarskjölds Väg 18, 751 85 Uppsala, Sweden; 2 Department of Clinical Sciences, Danderyd Hospital, Karolinska Institutet, Stockholm, Sweden; 3 Department of Neuroscience, Uppsala University, Uppsala, Sweden

**Keywords:** Cerebrospinal fluid, Targeted metabolomics, Flow injection analysis, High-resolution mass spectrometry, Secondary progressive multiple sclerosis

## Abstract

**Introduction:**

Standardized commercial kits enable targeted metabolomics analysis and may thus provide an attractive complement to the more explorative approaches. The kits are typically developed for triple quadrupole mass spectrometers using serum and plasma.

**Objectives:**

Here we measure the concentrations of preselected metabolites in cerebrospinal fluid (CSF) using a kit developed for high-resolution mass spectrometry (HRMS). Secondarily, the study aimed to investigate metabolite alterations in patients with secondary progressive multiple sclerosis (SPMS) compared to controls.

**Methods:**

We performed targeted metabolomics in human CSF on twelve SPMS patients and twelve age and sex-matched healthy controls using the Absolute IDQ-p400 kit (Biocrates Life Sciences AG) developed for HRMS. The extracts were analysed using two methods; liquid chromatography-mass spectrometry (LC-HRMS) and flow injection analysis-MS (FIA-HRMS).

**Results:**

Out of 408 targeted metabolites, 196 (48%) were detected above limit of detection and 35 were absolutely quantified. Metabolites analyzed using LC-HRMS had a median coefficient of variation (CV) of 3% and 2.5% between reinjections the same day and after prolonged storage, respectively. The corresponding results for the FIA-HRMS were a median CV of 27% and 21%, respectively. We found significantly (p < 0.05) elevated levels of glycine, asymmetric dimethylarginine (ADMA), glycerophospholipid PC-O (34:0) and sum of hexoses in SPMS patients compared to controls.

**Conclusion:**

The Absolute IDQ-p400 kit could successfully be used for quantifying targeted metabolites in the CSF. Metabolites quantified using LC-HRMS showed superior reproducibility compared to FIA-HRMS.

**Electronic supplementary material:**

The online version of this article (10.1007/s11306-020-1648-5) contains supplementary material, which is available to authorized users.

## Introduction

Non-targeted and targeted metabolomics methods provide a valuable tool for profiling low-weight molecules within biological samples, enabling enhanced understanding of disease pathology (Karakitsou et al. 2019). The major challenges in non-targeted metabolomics are typically related to metabolite identification (Viant et al. 2017), solving computationally data demanding tasks (Schrimpe-Rutledge et al. 2016) and standardization of protocols and methods to enable easily cross-institutional integration and comparison of data and results (Dias and Koal 2016). We and others have made substantial work to improve metabolite identification (Blaženović et al. 2018), computational solutions (Emami Khoonsari et al. 2019; Novella et al. 2019; Peters et al. 2019) and standardization of methods and protocols (Hoffmann et al. 2019). However, from a clinical point of view, any single or multi-panel based assay must rely on absolute quantifiable measures enabling extensive method validation, documentation and quality control. Standardized commercial kits enabling quantitative measures (based on full calibration curves) may provide an attractive complement to the more explorative non-targeted approaches. However, these kits are typically developed and validated for serum and plasma using traditional triple quadrupole mass spectrometry (QqQ-MS/MS) and only in a few cases have they been used for cerebrospinal fluid (CSF) (Koal et al. 2015; Varma et al. 2018).

Due to its proximity to the central nervous system (CNS), CSF is better for study diseases affecting the brain. Today, more than 450 metabolites have been identified and quantified in human CSF (Wishart et al. 2008) and several of these have been found altered in neurological diseases, such as Alzheimer's disease (Wilkins and Trushina 2018), Parkinson’s disease (Willkommen et al. 2018), Huntington’s disease (Herman et al. 2019b) and multiple sclerosis (MS) (Herman et al. 2018, 2019a; Reinke et al. 2014).

Multiple sclerosis is a chronic autoimmune and neurodegenerative disease that affect variable areas in the CNS, making the clinical presentation highly heterogeneous. Most of the patients are initially diagnosed with relapsing–remitting MS (RRMS), in which relapses or lesions cause neurological damage, followed by a complete or partial recovery. After a variable amount of time, many RRMS patients develop a later stage of the disease, called secondary progressive MS (SPMS). Secondary progressive MS is a neurodegenerative phenotype that typically is diagnosed retrospectively based on insidious development of disability and neuronal loss. Despite substantial efforts in drug development, treatment options for the later stage remains sparse. A characterization of the SPMS pathogenesis and its underlying molecular basis, which currently is largely unknown, could open a window of opportunity for future treatment development and improve patient stratification.

We have previously used non-targeted metabolomics methods of CSF to increase understanding of SPMS (Herman et al. 2019a) and provided a methodology to integrate multiple layers of information to improve early detection of transitioning patients (Herman et al. 2018).

Herein, we evaluated a recently developed kit for high-resolution mass spectrometry (HRMS) (Biocrates Life Science AG, Absolute IDQ-p400) that enable quantification of 408 metabolites in serum and plasma. This is a substantial increase in the number of metabolites quantifiable compared to the preceding kit (Absolute IDQ-p180) developed for QqQ-MS/MS that enabled quantification of 180 metabolites. We adapted this kit for the quantification of metabolites in human CSF and in a small, but well age and sex-matched, cohort of SPMS patients and controls used to explore the differences in metabolite concentrations. The kit has not been evaluated for use in CSF previously. In addition we also evaluate the impact of prolonged storage of prepared samples with respect to metabolite quantification for the included liquid chromatography-mass spectrometry (LC-HRMS) and flow injection analysis-MS (FIA-HRMS) methods. Finally, the concentration of detected metabolites are compared to what has been reported in the literature previously.

## Experimental

### Chemicals

Ethanol (≥ 99.5%) was purchased from CCS Healthcare (Malmö, Sweden), methanol (≥ 99.9%, MS grade) and pyridine (p.a. grade) from Honeywell (Seelze, Germany), acetonitrile (≥ 99.9%, MS grade) and isopropanol (≥ 99.9%, MS grade) from VWR Chemicals (Leuven, Belgium), formic acid (ACS reagent grade) from Merck (Darmstadt, Germany), and ammonium acetate (≥ 98%), phenylisothiocyanate (PITC; 99%, sequencing grade) and phosphate buffered saline (p.a. grade) from Sigma (Steinheim, Germany).

### Study cohorts and samples

Cerebrospinal fluid was collected from a cohort of MS patients (n = 12) and healthy controls (n = 12) in accordance with the ethical standards of the institutional and national research committee (Regional Ethical Board of Uppsala, Dnr 2008/182; Regional Ethical Board of Umeå, Dnr 08-157M) and with the 1964 Helsinki declaration and its later amendments or comparable ethical standards. Informed consent was obtained from all individual participants included in the study.

The clinical and demographic data of the patients and controls are summarized in Table 1. None of the MS patients were on any disease-modifying treatments. The samples were stored at − 80 °C until analysis.

**Table 1:** Clinical and demographic data of the patients and controls

Cohort	Study group	Control group
n	12	12
Age in years, mean (± SD)	58.7 (± 7.5)	54.2 (± 6.1)
Female/male	7/5	7/5
EDSS, median (range)	4.5 (3–6)	n/a
Disease duration in years, mean (± SD)	25.1 (± 11.8)	n/a

### Targeted CSF metabolomic analysis

A targeted metabolomic analysis was performed using the Absolute IDQ-p400 kit (Biocrates Life Science AG, Innsbruck, Austria), a commercially available assay which was originally developed for plasma, covering 408 metabolites from eight metabolite classes. The largest metabolite class that was covered was the glycerophospholipids (n = 196) followed by glycerides (n = 60), acylcarnitines (n = 55), sphingolipids (n = 40), amino acids (n = 21), biogenic amines (n = 21), cholesteryl esters (n = 14) and monosaccharides (n = 1) which included the sum of hexoses (including glucose).

The LC-HRMS method was used to quantify amino acids and biogenic amines while acylcarnitines, cholesterol esters, glycerophospholipids, glycerides, sphingolipids and hexoses were assessed using FIA-HRMS. The kit provided quantitative measurements for twelve acylcarnitines, 21 amino acids, 21 biogenic amines and the sum of hexoses. For the amino acids and biogenic amines, standard solutions for calibration curves at six levels including isotopically labelled internal standards were provided for each metabolite. Five of the compounds in these two classes were quantified with restriction, meaning that the calibration curves had expected coefficients of determination (R^2^) < 0.99 according to the manufacturer of the kit. For the acylcarnitines and sum of hexoses, standards at one concentration were provided (one-point calibration using FIA). The rest of the metabolites were measured semiquantitatively, e.g. that standards with similar chemical properties as the targets were used (a version of one-point calibration). The names of metabolites of the following subclasses were annotated according to the following: acylcarnitines (AC), diglycerides (DG), triglycerides (TG), lysophosphatidylcholines (LPC), phosphatidylcholines (PC), sphingomyelins (SM), ceramides (Cer) and cholesteryl esters (CE).

The frozen CSF samples (− 80 °C) were placed on ice up until completely thawed then centrifuged at 2750×*g* for 5 min at 4 °C. According to recommendations from the kit manufacturer, the sample volume used for CSF was 30 µL, instead of 10 µL used for plasma in the kit protocol, since the concentrations of most metabolites are lower in CSF compared with plasma. This is the same sample volume that has been used for CSF in studies using the preceding kit Absolute IDQ-p180 (Koal et al. 2015; Mandal et al. 2012). Sample preparation was performed according to the protocol that was provided with the kit. Briefly, the samples were prepared on the kit plate (all plates provided with the kit were in the 96-well format), by first adding stable isotope–labelled standards followed by 30 µL CSF. The samples were then dried using an evaporator, and subsequently derivatized by the addition of 50 µL of a 5% solution of phenylisothiocyanate (in water:ethanol:pyridine, 1:1:1) followed by incubation in room-temperature for 20 min. The samples were then dried again, and extracted by the addition of 300 µL 5 mM ammonium acetate in methanol (MeOH) and shaking at 450 rpm for 30 min. The extracts were collected by centrifugation into the provided collection plate. For LC-HRMS analysis, 150 µL of the samples were transferred and diluted with 150 µL H_2_O on an empty plate, and for FIA-HRMS analysis, 250 µL of the FIA mobile phase (made by mixing 290 mL MeOH and a 10 mL ampule Biocrates FIA mobile phase additive, provided with the kit) were added directly to the samples on the collection plate.

The extracts were analysed using an Ultimate 3000 HPLC (Thermo Scientific™) coupled to a high-resolution Q Exactive™ hybrid quadrupole-Orbitrap mass spectrometer (Thermo Scientific™) using electrospray ionization. The instrumental analysis was performed according to the guidelines from the manufacturer. Briefly, the analysis were done in the positive and negative ionization modes for both LC-HRMS and FIA-HRMS, with data collection in the fullscan (MS1) mode. The source parameters used for the LC-HRMS analysis were: sheath gas flow rate 60, auxiliary gas flow rate 30, sweep gas flow rate 1, spray voltage 3 kV, capillary temperature 300 °C, S-lens RF level 60, and auxiliary gas heater temperature 500 °C; and for FIA-HRMS: sheath gas flow rate 15, auxiliary gas flow rate 5, sweep gas flow rate 1, spray voltage 2.5 kV, capillary temperature 300 °C, S-lens RF level 60, and auxiliary gas heater temperature 120 °C. The chromatography was done using the UHPLC column provided with the kit (available only from Biocrates), with the injection volume 5 µL. Mobile phase A was 0.2% formic acid in H_2_O and mobile phase B 0.2% formic acid in ACN. The chromatographic program was 6 min, including a gradient from 0 to 95% B over 4 min, followed by washing (95% B) and equilibration (0% B). The flow rate was 0.8 mL/min and the column oven temperature 50 °C. The FIA analysis was done by injecting 20 µL of the sample into the flow of the FIA program, lasting for 5 min and pumping the FIA mobile phase (10 mL ampule Biocrates FIA mobile phase additive in 290 mL MeOH) at the flow rate 0.05 mL/min for the first 1.6 min and then increasing the flow rate to 0.2 mL/min for 1.2 min and then back to 0.05 mL/min for the rest of the program.

The measurements were made in two batches using a starter kit in a 96-well format for a total of 24 samples. Each batch included twelve samples that were evenly balanced with regards to disease, sex and age, six calibration standards and two quality control samples. The highest calibration point and highest quality control sample provided with the kit were not used since the metabolite concentration in CSF is much lower than in plasma. The data were preprocessed using the MetIDQ software (Biocrates, Life Science AG) prior to statistical evaluation. The final concentrations of metabolites that were quantified using full calibration curves (amino acids and biogenic amines) and one-point calibration (twelve acylcarnitines and sum of hexoses) were compared with values found in the CSF metabolome database (adult subjects only, Wishart et al. 2008) and the experimental and literature values reported by Mandal et al. (2012), who used the preceding kit Absolute IDQ-p180 developed for QqQ-MS/MS. The remaining semiquantitatively metabolite levels cannot be considered reliable quantitative measures, but they were included in the statistical analysis.

To estimate within-day variability, the second batch of samples were injected twice immediately after one another. To evaluate sample long-term stability, the prepared samples of the same batch were reinjected after storage at 4 °C for an extended time; for the LC-HRMS method, samples were stored for five days, whereas samples for the FIA-HRMS method were stored for seven days. The coefficient of variance (CV) was calculated within-subject between immediate reinjections to estimate within-day variability, and between the first injection and the reinjection after storage to estimate long-term stability. The median of within-subject CVs for each metabolite was then computed.

### Statistical analysis

The data were imported into the statistical software environment R v3.6.0 (“R: The R Project for Statistical Computing” n.d.) and metabolites that were not present in at least 75% of the samples were removed. The concentrations were log_2_ transformed and linear models were trained for each metabolite (using the *lm* function from the R package *stats*), with age, sex, diagnostic condition (SPMS/control) and batch assignment, as predictors. The same analyses were also repeated excluding sex as a covariate since this variable had little impact.

## Results

### Profiling of the CSF metabolome

**Fig. 1:**
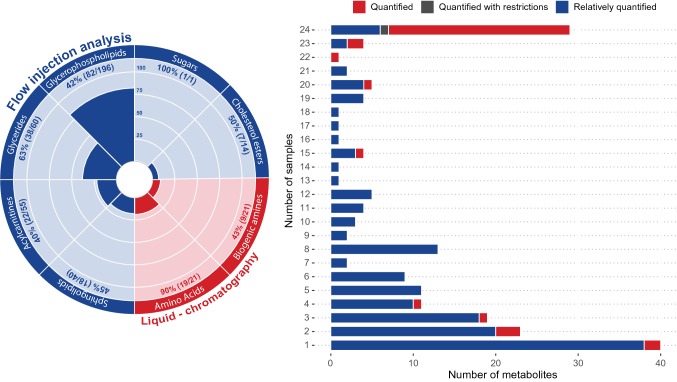
The polar histogram (left) illustrates the number of detected compounds from the eight metabolite classes. Flow injection HRMS (in blue) was used to analyse 90% of the compounds, while amino acids and biogenic amines (in red) were analysed by LC-HRMS. The stacked plot (right) shows the number of detected metabolites (x-axis) *versus* the number of samples in which they were detected (y-axis). The metabolites are coloured according to the quantification method: absolute quantification (red), quantification with restriction (grey) and relative quantification (blue)

Out of 408 targeted metabolites, 196 (48%) were detected above the limit of detection (LOD; defined for each metabolite as three times the background noise level), corresponding to 82 glycerophospholipids, 38 glycerides, 22 acylcarnitines, 19 amino acids, 18 sphingolipids, 9 biogenic amines, 7 cholesterol esters and the sum of hexoses (Fig. 1). Thirty compounds were quantified in all samples; 23 of these compounds were absolutely quantified, one was quantified with restriction and six were relatively quantified. Forty compounds were only detected once over the set of 24 samples (Fig. 1).

### Quantification of CSF metabolites

The kit enabled absolute quantification of 55 metabolites, of which 35 were observed above the LOD in at least one CSF sample. The results are summarized in Table 2, including mean and range concentration (in µM) for each metabolite, as well as the number of samples in which they were quantified. All amino acids were quantified in all samples except xleucine (sum of leucine and isoleucine), tyrosine, isoleucine, asparagine, glutamine and proline. Five biogenic amines (creatinine, taurine, t4-OH-Pro, SDMA and ADMA) and two acylcarnitines (AC (0:0) and AC (2:0)) were quantified in all samples respectively. The quantified concentrations were compared with the reference values for CSF metabolites provided by Mandal et al. (2012) and the CSF metabolome database (adult subjects only, Wishart et al. 2008) (Table 2). The present result shows a good agreement with the experimental and literature values reported by Mandal et al. (annotated Mandal 1 and 2, respectively, in Table 2) who used the preceding kit Absolute IDQ-p180 for targeted metabolomics. The expanded IDQ-p400 kit, enabled absolute quantification (full calibration curve or one-point calibration) of metabolites that not have been reported in CSF previously in the CSF metabolome database or by Mandal et al. These included the acylcarnitines: tetradecanoylcarnitine (AC (14:0)) and octadecanoylcarnitine (AC (18:0)), and biogenic amines: asymmetric dimethylarginine (ADMA), nitrotyrosine, putrescine, symmetric dimethylarginine, spermidine and trans-4-hydroxyproline. The mean concentration of the metabolites that were quantified using relative one-point calibration are presented in Supplemental Table 1.

**Table 2:** A summary of the 35 metabolites that were absolutely quantified in the analyzed cohort

Metabolite	Class	Mean (± SD)	Range	n	*Mandal 1*	*Mandal 2*	*CSF MDB*
		(µM)	(µM)		Mean (± SD) (µM)	Mean (± SD) (µM)	Mean (n)
Carnitine (AC (0:0))	Acylcarnitines	1.69 (± 0.263)	1.34–2.46	24	1.900 (± 0.5)	4.0 ± 2.0	–
Acetylcarnitine (AC (2:0)) b	Acylcarnitines	0.407 (± 0.158)	0.182–0.794	24	0.322 (± 0.148)	–	–
Butyrylcarnitine (AC (4:0))	Acylcarnitines	0.136 (± 0.003)	0.134–0.138	2	0.024 (± 0.007)	–	–
Valerylcarnitine (AC (5:0))	Acylcarnitines	0.079 (± 0.003)	0.077–0.083	3	0.013 (± 0.006)	–	–
Tetradecanoylcarnitine (AC (14:0))	Acylcarnitines	0.058 (± 0.005)	0.055–0.062	2	–	–	–
Octadecanoylcarnitine (AC (18:0))	Acylcarnitines	0.024	–	1	–	–	–
Alanine	Amino acids	32.7 (± 7.07)	21.6–49.1	24	46 (± 27)	37 ± 7	7.8 (3)
Arginine	Amino acids	22.5 (± 3.48)	16.8–29.6	24	17.9 (± 5.7)	20.2 ± 6.3	21.6 (3)
Asparagine	Amino acids	6.6 (± 1.33)	5.03–11.1	24	4 (± 2)	5 ± 1	3.89 (2)
Citrulline b	Amino acids	1.93 (± 0.593)	0.93–3.32	24	–	–	16.19 (2)
Glutamine	Amino acids	473 (± 37.9)	400–546	24	398 (± 150)	444 ± 80	700.3 (2)
Glycine b,c	Amino acids	6.07 (± 1.51)	2.91–10.1	24	6.1 (± 1.4)	8.2 ± 3.0	3.76 (3)
Histidine	Amino acids	12.6 (± 1.78)	8.91–15.6	24	15 (± 8)	12 ± 2	72.2 (2)
Isoleucine	Amino acids	6.63 (± 0.913)	5.75–7.68	4	7 (± 5)	8 ± 3	4.2 (2)
Lysine a	Amino acids	23.8 (± 3.43)	14.5–30.9	24	29 (± 13)	28 ± 8	44.6 (1)
Methionine b	Amino acids	4.27 (± 2.15)	2.4–12.1	24	5 (± 4)	6 ± 3	2.7 (1)
Ornithine d	Amino acids	4.28 (± 1.02)	2.65–7.3	24	4.5 (± 2.2)	6.0 ± 1.5	288 (1)
Phenylalanine a	Amino acids	8.77 (± 1.73)	5.81–13.4	24	15 (± 8)	18 ± 7	10.94 (5)
Proline	Amino acids	0.952 (± 0.539)	0.432–2.06	15	1.9 (± 1)	4.0 ± 2.0	0.68 (1)
Serine	Amino acids	23.9 (± 4.38)	15.8–32.2	24	38 (± 14)	42 ± 15	18.75 (2)
Threonine	Amino acids	27.3 (± 5.29)	12.5–35.2	24	28 (± 8)	28 ± 5	9.68 (1)
Tryptophan	Amino acids	1.77 (± 0.363)	1.03–2.61	24	10 (± 5)	2 ± 1	3.29 (14)
Tyrosine	Amino acids	8.38 (± 1.86)	4.65–13.1	22	12 (± 9)	10 ± 4	13.783 (10)
Valine a,c	Amino acids	16.4 (± 3.84)	8.89–23.4	24	19 (± 13)	24 ± 7	51.37 (2)
Leucine + Isoleucine a	Amino acids	16.5 (± 3.53)	8.68–22.5	23	16 (± 9)	19 ± 4	Leu: 11.5 (1)
Asymmetric dimethylarginine b,c	Biogenic amines	0.058 (± 0.016)	0.031–0.093	24	–	–	–
Creatinine	Biogenic amines	64.9 (± 12)	47.9–94.2	24	44 (± 13)	–	–
Kynurenine b	Biogenic amines	0.066 (± 0.017)	0.041–0.101	20	–	–	1.16 (4)
Nitrotyrosine	Biogenic amines	0.136	–	1	–	–	–
Putrescine	Biogenic amines	0.14 (± 0.042)	0.071–0.246	23	–	–	–
Symmetric dimethylarginine	Biogenic amines	0.128 (± 0.028)	0.09–0.2	24	–	–	–
Spermidine	Biogenic amines	0.104 (± 0.005)	0.1–0.107	2	–	–	–
*trans*-4-Hydroxyproline	Biogenic amines	0.563 (± 0.222)	0.247–1.16	24	–	–	–
Taurine	Biogenic amines	6.96 (± 1.97)	4.09–12.8	24	–	–	5.76 (3)
Hexoses (including glucose) c	Sugars	3790 (± 530)	3031–5272	24	2960 (± 1110)	5390 ± 1650	3700 (1)

The mean concentrations, standard deviations, concentration ranges and the number of samples in which the metabolites were detected are presented, as well as reference values from (Mandal et al. 2012) and the CSF metabolome database (Wishart et al. 2008).

*Mandal 1*: Experimental values from Mandal et al 2012; *Mandal 2*: Literature values from Mandal et al 2012; *CSF MDB*: Experimental values extracted from the CSF metabolome database, quantitative CSF data extracted for adult subjects only with number of entries in the database within parentheses.

^a,b,c^See Table 3: ^a^Affected by age, ^b^Affected by batch, ^c^Affected by diagnosis, ^d^Orn was quantified with restriction, see the Experimental section.

**Table 3:** Metabolites significantly associated to participant age, sample batch and/or altered in SPMS patients compared with controls with corresponding p-values as well as their within-day reproducibility and long-term stability estimated with CV

Metabolite	Class	Analysis method	Quantification	Age (p-value)	Batch (p-value)	SPMS vs control (p-value)	CV (%) (within)	CV (%) (between)	n (CV)
AC (0:0)	Acylcarnitines	FIA	Quantitative	n.s	n.s	n.s	3	9	12
AC (14:1)	Acylcarnitines	FIA	Relative Quantitative	n.s	< 0.001	n.s	11	46	3
AC (2:0)	Acylcarnitines	FIA	Quantitative	n.s	0.015	n.s	13	24	12
AC (4:0-DC)	Acylcarnitines	FIA	Relative Quantitative	n.s	< 0.001	n.s	28	18	8
Alanine	Amino acids	LC	Quantitative	n.s	n.s	n.s	4	3	12
Arginine	Amino acids	LC	Quantitative	n.s	n.s	n.s	1	1	12
Asparagine	Amino acids	LC	Quantitative	n.s	n.s	n.s	2	2	12
Citrulline	Amino acids	LC	Quantitative	n.s	0.017	n.s	4	3	10
Glutamine	Amino acids	LC	Quantitative	n.s	n.s	n.s	2	2	12
Glycine	Amino acids	LC	Quantitative	n.s	0.020	0.016	2	2	12
Histidine	Amino acids	LC	Quantitative	n.s	n.s	n.s	1	1	12
Isoleucine	Amino acids	LC	Quantitative	–	–	–	8	13	4
Lysine	Amino acids	LC	Quantitative	0.017	n.s	n.s	2	2	12
Methionine	Amino acids	LC	Quantitative	n.s	0.009	n.s	2	1	12
Ornithine	Amino acids	LC	Quantitative with Restrictions	n.s	n.s	n.s	3	3	12
Phenylalanine	Amino acids	LC	Quantitative	0.040	n.s	n.s	3	3	12
Serine	Amino acids	LC	Quantitative	n.s	n.s	n.s	2	1	12
Threonine	Amino acids	LC	Quantitative	n.s	n.s	n.s	1	1	12
Tryptophan	Amino acids	LC	Quantitative	n.s	n.s	n.s	7	11	12
Tyrosine	Amino acids	LC	Quantitative	n.s	n.s	n.s	1	1	11
Valine	Amino acids	LC	Quantitative	0.008	n.s	n.s	5	3	12
xLeucine	Amino acids	LC	Quantitative	0.001	n.s	n.s	3	3	12
ADMA	Biogenic amines	LC	Quantitative	n.s	< 0.001	0.009	9	5	12
Creatinine	Biogenic amines	LC	Quantitative	n.s	n.s	n.s	7	8	12
Kynurenine	Biogenic amines	LC	Quantitative	n.s	0.007	n.s	–	–	–
Putrescine	Biogenic amines	LC	Quantitative	n.s	n.s	n.s	7	7	9
SDMA	Biogenic amines	LC	Quantitative	n.s	n.s	n.s	3	4	12
t4-OH-Pro	Biogenic amines	LC	Quantitative	n.s	n.s	n.s	3	2	12
Taurine	Biogenic amines	LC	Quantitative	n.s	n.s	n.s	2	2	12
CE (18:1)	Cholesterol esters	FIA	Relative Quantitative	n.s	0.009	n.s	73	70	6
CE (18:2)	Cholesterol esters	FIA	Relative Quantitative	n.s	0.033	n.s	79	89	9
CE (20:4)	Cholesterol esters	FIA	Relative Quantitative	–	–	–	8	12	7
TG (50:2)	Glycerides	FIA	Relative Quantitative	–	–	–	35	30	4
TG (52:4)	Glycerides	FIA	Relative Quantitative	n.s	n.s	n.s	71	112	11
TG (53:4)	Glycerides	FIA	Relative Quantitative	n.s	n.s	n.s	13	50	5
PC (29:1)	Glycerophospholipids	FIA	Relative Quantitative	–	–	–	68	40	4
PC (32:0)	Glycerophospholipids	FIA	Relative Quantitative	n.s	n.s	n.s	14	11	12
PC (34:1)	Glycerophospholipids	FIA	Relative Quantitative	n.s	n.s	n.s	6	5	12
PC (34:2)	Glycerophospholipids	FIA	Relative Quantitative	n.s	n.s	n.s	13	10	12
PC (36:1)	Glycerophospholipids	FIA	Relative Quantitative	–	–	–	14	15	12
PC (36:2)	Glycerophospholipids	FIA	Relative Quantitative	n.s	n.s	n.s	13	3	3
PC (38:5)	Glycerophospholipids	FIA	Relative Quantitative	–	–	–	13	21	5
PC (40:3)	Glycerophospholipids	FIA	Relative Quantitative	n.s	n.s	n.s	42	82	6
PC (40:6)	Glycerophospholipids	FIA	Relative Quantitative	n.s	n.s	n.s	27	10	5
PC (44:5)	Glycerophospholipids	FIA	Relative Quantitative	n.s	n.s	n.s	46	19	9
PC-O (31:1)	Glycerophospholipids	FIA	Relative Quantitative	n.s	< 0.001	n.s	–	–	–
PC-O (33:0)	Glycerophospholipids	FIA	Relative Quantitative	n.s	n.s	n.s	27	47	8
PC-O (34:0)	Glycerophospholipids	FIA	Relative Quantitative	n.s	0.024	0.046	28	21	9
PC-O (34:2)	Glycerophospholipids	FIA	Relative Quantitative	–	–	NA	85	43	9
PC-O (36:2)	Glycerophospholipids	FIA	Relative Quantitative	n.s	0.012	n.s	–	–	–
SM (34:1)	Sphingolipids	FIA	Relative Quantitative	n.s	0.008	n.s	34	33	12
Hexoses	Sugars	FIA	Quantitative	n.s	n.s	0.010	2	2	12

*n.s.* non-significant

### Stability of prepared samples following storage at 4 °C

To explore the metabolites within-day reproducibility and their long-term stability, the second batch was injected twice at analysis and reinjected after five (LC-HRMS) or seven (FIA-HRMS) days of storage in 4 °C. 36 metabolites demonstrated a median CV < 20% between reinjections on the same day out of which 32 of these still demonstrated median CVs < 20% after prolonged storage (Table 3). Comparing the analytes measured using the LC-HRMS method with those measured with FIA-HRMS revealed that the LC-HRMS method was more stable with a median CV of 3% compared with 27% for the FIA-HRMS between immediate reinjections. This trend remained after prolonged storage where the median CV of LC analytes was estimated at 2.5% and 21% for the FIA analytes, respectively.

### Metabolite associations to age, batch and disease effect

**Fig. 2:**
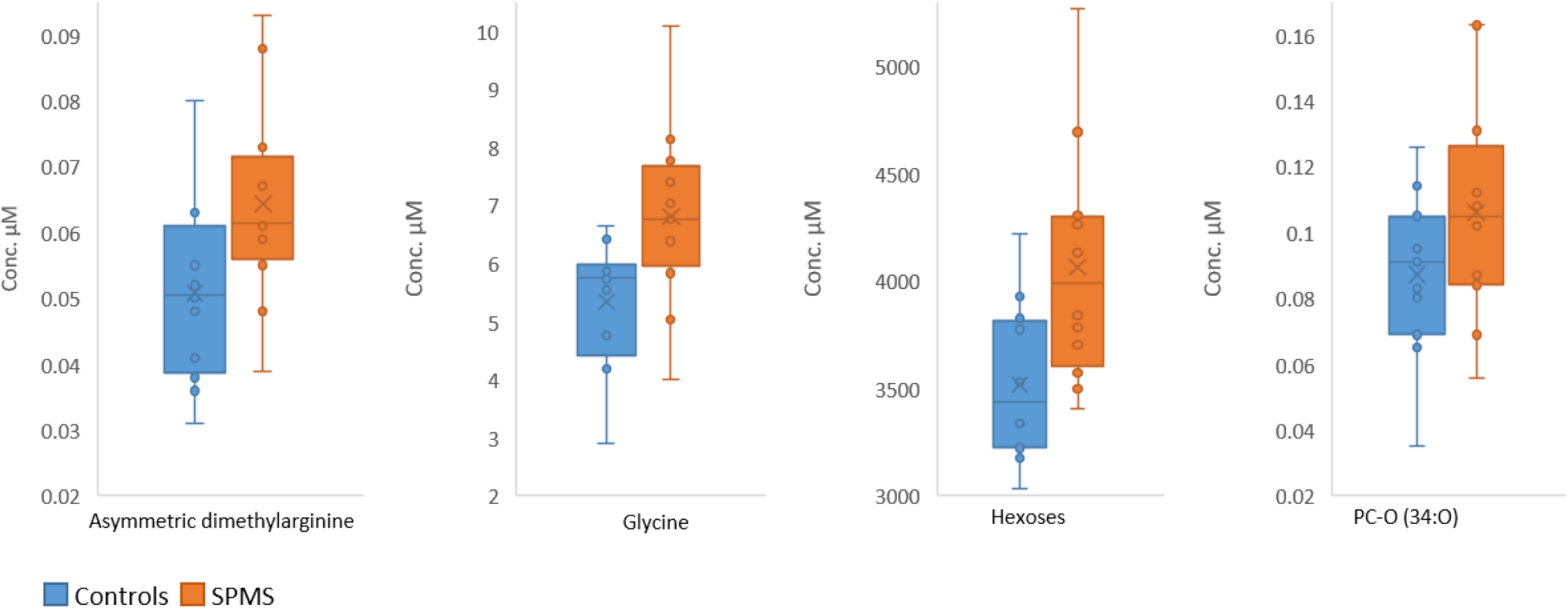
Boxplots of asymmetric dimethylarginine, glycine, hexoses and PC-O (34:0) which were significantly (p < 0.05) elevated in SPMS patients compared with the controls. The middle line and x of the boxes represents the median and mean concentrations, respectively. The bottom and top lines of the boxes represent the first and third quartiles, respectively. The whiskers indicate the lowest and highest metabolite concentrations of the groups

To assess the effect of age and sex of participants, as well as batch assignment and to explore differences in metabolite concentrations in patients with SPMS compared to age and sex-matched healthy controls, linear models were trained on the log_2_ transformed concentrations of each of the 46 metabolites that were present in at least 75% of the samples. Taurine was the only metabolite affected by sex (p = 0.004, not included in Table 3). After removing sex as a covariate, significantly affected metabolites (p-value < 0.05) are listed in Table 3 (full list of metabolites are presented in Supplemental Table 2). Four amino acids were significantly affected by age (lysine, phenylalanie, valine and xleucine), 14 metabolites by batch assignments (AC (2:0), AC (4:0-DC), AC (14:1), citrulline, glycine, methionine, ADMA, kynurenine, PC-O (31:1), PC-O (34:0), PC-O (36:2), SM (34:1), CE (18:1) and CE (18:2)), and four metabolites (glycine, ADMA, PC-O (34:0) and hexose) were elevated in SPMS patients compared to controls (Fig. 2).

## Discussion

Metabolites in the CSF were profiled using the Absolute IDQ-p400 kit for targeted metabolomics. To our knowledge, this is the first study to utilize this kit for CSF. Of the 408 metabolites present in the kit, 196 (48%) of the targeted metabolites were successfully quantified (48%). Previously this kit has been used for lipidomic analyses in liver samples (Kanemoto et al. 2019) and to investigate possible circulating markers of pancreatic fat in plasma (Jaghutriz et al. 2019; Kanemoto et al. 2019). The preceding kit Absolute IDQ-p180 developed for QqQ-MS, targeting a smaller number of metabolites (n = 180), has previously been used for characterization of the CSF metabolome (Koal et al. 2015; Mandal et al. 2012; Illig et al. 2010). Mandal et al. (2012) quantified 78 metabolites in CSF using a 4000 QTrap instrument, while a study conducted by the manufacturer of the kit (Biocrates, Life Science AG, Application Note 1003-1) quantified 65 metabolites in pooled CSF using the same setup. Herein we demonstrated that by using the expanded kit utilizing HRMS, the coverage of quantified metabolites can be increased by a factor greater than two. The CSF levels of the overlapping metabolites with the preceding kit were in agreement with previous measurements from Mandal et al., the manufacturer of the kit (Biocrates) and with the CSF metabolome database. Here, we also report quantification of a number of metabolites that, to our knowledge, have not been reported in CSF before. These included the acylcarnitines:tetradecanoylcarnitine (AC (14:0)) and octadecanoylcarnitine (AC (18:0)), and biogenic amines: ADMA, nitrotyrosine, putrescine, symmetric dimethylarginine (SDMA), spermidine and trans-4-hydroxyproline. Asymmetric dimethylarginine was especially interesting as it was significantly elevated in SPMS patients compared to controls, which previously has been shown to also be the case in comparison to other neurological diseases (Haghikia et al. 2015). The concentrations of metabolites measured using relative one-point calibration are not discussed any further at this point since this cannot be considered a reliable measure of quantification.

Out of the 196 detected metabolites, it was possible to estimate within-day and long-term stability for 49 metabolites. Here 36 metabolites demonstrated reasonable CV (CV < 20%) after reinjections on the same day, where 32 of these were also stable after prolonged storage. The amino acids and biogenic amines that were analysed using the LC-HRMS method showed clearly better within and between days variation (median CV 3% and 3%) compared to compounds analysed by the FIA-HRMS method (median CV 27% and 21%). Based on these results many LC analytes seem to be stable for at least five days while stored at 4 °C, which allows for reinjection, larger injection queues and follow-up experiments of these analytes. The large variation observed for the FIA-HRMS method is most likely due to methodological aspects, such as ion suppression and matrix effects using no chromatographic separation. The median within-day CV of 27% for the FIA analytes should be considered high when performing targeted analysis. Furthermore, to obtain reliable results several reinjections may be required. We would suggest careful evaluation of the robustness and reliability of the FIA analytes prior to application in large-scale studies.

The remaining metabolites were either below the LOD in the second batch or were not detected in either of the three injections (the two injections within the same day and the delayed injection). Proline for example was detected in 15/24 samples of which only three samples were in the second batch and none of these were above LOD in the immediate reinjections or delayed injections. Hence, the within-day reproducibility and long-term stability could not be estimated for proline. TG (48:2) which was detected in 19/24 samples out of which ten were in the second batch, was on the other hand detected in the first and immediately repeated injections, but not detected after prolonged storage.

According to recommendations from the manufacturer of the kit, prepared samples for the LC part can be stored up to two days at 4 °C and samples for the FIA part up to seven days. Here we show that the metabolites measured using LC can be stored for at least five days at 4 °C. In the FIA part we did not see an increased CV, but some metabolites were detected to lesser content. The possible impact of freeze-thawing of derivatized samples should typically be evaluated in a systematic manner for each metabolite measured (He et al. 2016). According to the manufacturer, the prepared samples should never be stored below 0 °C, thus making it challenging to store prepared samples long-term and for batching larger number of samples to be measured simultaneously.

Linear models were used to investigate the effect of age, batch assignment and SPMS (*versus* healthy controls) on the log_2_ quantified concentrations of CSF metabolites. Four metabolites were found to be affected by the disease and four others by age. Glycine, asymmetric dimethylarginine, PC-O (34:0) and hexoses were all significantly elevated in SPMS patients compared with controls. Elevated glycine levels in CSF have previously been observed in multiple sclerosis patients, possibly due to oxidative stress (Ďurfinová et al. 2018; Haghikia et al. 2015), whereas altered hexose/glucose metabolism could potentially be of importance for multiple sclerosis as it has been implicated in other neurodegenerative disorders (Pathak et al. 2013). The increased levels of the glycerophospholipid PC-O (34:0) in SPMS patients has to our knowledge not been demonstrated previously, but altered levels of lipid composition in multiple sclerosis patients has recently been found in other cohorts of patients also (Nogueras et al. 2019; Pathak et al. 2013).

Lysine, phenylalanine, valine and xleucine demonstrated significant associations to age. Linear age associations have previously been demonstrated for all of these metabolites (including leucine and isoleucine separately) in blood (Menni et al. 2013) and now also in CSF. Valine and xleucine are both from the same metabolic sub-pathway *valine, leucine and isoleucine metabolism* which according to the study by Menni et al. contain eleven metabolites that demonstrated highly significant age dependencies in fasting blood.

This study contained a couple of limitations. The most prominent limitation was the small cohort size, which was due to the study being a pilot study and the challenge of collecting CSF from healthy subjects, which is rarely done. This was of course also limiting the power of the performed statistical tests and hence the strength of the biological findings. Therefore, all biological findings herein needs to be reproduced in a larger cohort. The study also lacked information regarding the subjects BMI and body weight, which has been shown to be linked to MS. On the other hand, the cohort was well-balanced in terms of age and sex between the SPMS patients and control subjects and no patients were on any disease-modifying treatments.

The Absolute IDQ-p400 kit should according to the manufacturer only be used for research purposes and not for diagnostic procedures. As a research tool, the sample preparation is relatively fast and straightforward and both the LC-HRMS and FIA-HRMS methods have short analytical procedures and results can be compared across institutions. However, a potential disadvantage would be the challenge of analysing large number of samples without storing prepared samples more than five to seven days.

## Conclusions

Using the Absolute IDQ-p400 kit developed for HRMS we could successfully quantify 196 targeted metabolites in the CSF out of which 35 metabolites could be absolutely quantified. These metabolites comprised 48% of the total number of metabolites in the kit. In terms of reproducibility and stability, metabolites measured using the LC-HRMS showed better reproducibility compared to those measured using the FIA-HRMS method. We also conclude that the majority of metabolites could be quantified after prolonged storage at 4 °C. The long term stability should however be further evaluated, as it could be a limiting factor when batching and analysing large number of samples and in case of re-analysis of prepared samples.

## Electronic supplementary material

Below is the link to the electronic supplementary material.

Supplementary file1 (PDF 304 kb)

## Data Availability

The metabolomics and metadata reported in this paper are available via MetaboLights (https://www.ebi.ac.uk/metabolights/MTBLS1279) study identifier MTBLS1279.
