# Supplementary material for: Targeted metabolomics of CSF in healthy individuals and patients with secondary progressive multiple sclerosis using high-resolution mass spectrometry
Source: Metabolomics. 2020 Feb 12;16(2):26. doi: 10.1007/s11306-020-1648-5 (PMC7015966; doi:10.1007/s11306-020-1648-5)
Supplement: Supplementary file 1 — Supplementary file1 (PDF 304 kb) [file 11306_2020_1648_MOESM1_ESM.pdf]

Supplementary information:

Targeted metabolomics of CSF in healthy individuals and patients with secondary progressive multiple sclerosis using high-resolution mass spectrometry

Henrik Carlsson[1], Sandy Abujrais[1], Stephanie Herman[1], Payam Emami Khoonsari[1], Anders Svenningsson[2] , Joachim Burman[3], Kim Kultima[1]

1) Department of Medical Sciences, Clinical Chemistry, Uppsala University, Uppsala, Sweden
2) Department of Clinical Sciences, Danderyd Hospital, Karolinska Institutet, Stockholm, Sweden
3) Department of Neuroscience, Uppsala University, Uppsala, Sweden

Contents of supporting information:

Table S1: The mean concentration (µM), standard deviation and concentration range of metabolites in CSF that were relatively quantified in the cohort of 12 SPMS patients and 12 controls.

Table S1. The mean concentration (μM), standard deviation and concentration range of metabolites in CSF that were relatively quantified in the cohort of 12 SPMS patients and 12 controls.

Metabolite	Class	Mean (±SD) conc. (μM)	Min-max conc. (μM)	n
AC(4:0-OH)	acylcarnitines	0.148(±0.02)	0.134-0.163	2
AC(4:1)	acylcarnitines	0.046(±0)	0.046-0.046	1
AC(4:1-DC)	acylcarnitines	0.008(±0.004)	0.003-0.014	8
AC(5:1)	acylcarnitines	0.091(±0.016)	0.073-0.101	3
AC(5:1-DC)	acylcarnitines	0.026(±0.005)	0.022-0.029	2
AC(7:0)	acylcarnitines	0.162(±0)	0.162-0.162	1
AC(7:0-DC)	acylcarnitines	0.131(±0.056)	0.07-0.181	4
AC(8:1)	acylcarnitines	0.086(±0.015)	0.064-0.114	8
AC(10:2)	acylcarnitines	0.011(±0)	0.011-0.011	1
AC(10:3)	acylcarnitines	0.015(±0.003)	0.013-0.019	4
AC(12:1)	acylcarnitines	0.13(±0.147)	0.045-0.299	3
AC(14:1-OH)	acylcarnitines	0.106(±0.014)	0.091-0.135	8
AC(14:2)	acylcarnitines	0.002(±0)	0.002-0.002	2
AC(19:0)	acylcarnitines	0.04(±0)	0.04-0.04	1
PC(32:0)	glycerophospholipids	0.261(±0.061)	0.173-0.397	24
PC(34:1)	glycerophospholipids	1.77(±0.462)	1.06-2.8	24

PC(34:2)	glycerophospholipids	0.231(±0.163)	0.133-0.959	24
SM(34:1)	sphingolipids	0.336(±0.108)	0.182-0.624	24
CE(18:2)	cholesterol esters	9.24(±10)	0.238-34.7	24
LPC(16:0)	glycerophospholipids	0.15(±0.239)	0.027-0.737	8
LPC(18:0)	glycerophospholipids	0.044(±0.018)	0.03-0.063	3
LPC(18:1)	glycerophospholipids	1.28(±0.778)	0.728-2.43	4
LPC(20:1)	glycerophospholipids	0.135(±0)	0.135-0.135	1
LPC-O(17:1)	glycerophospholipids	0.175(±0)	0.175-0.175	1
PC(25:0)	glycerophospholipids	0.198(±0.068)	0.094-0.335	14
PC(29:0)	glycerophospholipids	0.248(±0.013)	0.231-0.26	4
PC(29:1)	glycerophospholipids	0.106(±0.111)	0.018-0.389	11
TG(52:4)	Glycerides	0.149(±0.103)	0.05-0.587	24
PC(30:3)	glycerophospholipids	0.492(±0)	0.492-0.492	1
PC(31:0)	glycerophospholipids	0.077(±0)	0.077-0.077	1
PC(31:1)	glycerophospholipids	0.123(±0.094)	0.037-0.223	3
PC(31:2)	glycerophospholipids	0.009(±0)	0.008-0.009	2
AC(14:1)	Acylcarnitines	0.069(±0.03)	0.023-0.131	23
PC(32:1)	glycerophospholipids	0.221(±0.003)	0.219-0.225	3
PC(32:2)	glycerophospholipids	0.142(±0)	0.142-0.142	1
PC(32:3)	glycerophospholipids	0.086(±0)	0.086-0.086	1

PC(33:2)	glycerophospholipids	0.02(±0)	0.02-0.02	1
PC(34:3)	glycerophospholipids	0.084(±0.025)	0.04-0.113	6
PC(34:5)	glycerophospholipids	2.12(±0.459)	1.5-2.77	8
PC(35:0)	glycerophospholipids	0.134(±0.052)	0.087-0.234	8
PC(35:2)	glycerophospholipids	0.052(±0.007)	0.045-0.059	3
PC(36:1)	glycerophospholipids	0.202(±0.06)	0.13-0.329	12
PC-O(34:0)	glycerophospholipids	0.097(±0.031)	0.035-0.163	23
PC(36:4)	glycerophospholipids	0.202(±0.048)	0.168-0.288	5
PC(36:6)	glycerophospholipids	0.034(±0.021)	0.012-0.078	8
PC(37:1)	glycerophospholipids	0.358(±0.008)	0.349-0.365	3
PC(38:1)	glycerophospholipids	10.9(±0.081)	10.8-10.9	2
PC(38:5)	glycerophospholipids	0.083(±0.044)	0.044-0.174	10
PC(38:6)	glycerophospholipids	0.102(±0.089)	0.045-0.204	3
PC(38:7)	glycerophospholipids	0.051(±0.009)	0.044-0.057	2
PC(40:1)	glycerophospholipids	0.083(±0.008)	0.077-0.092	3
PC(40:2)	glycerophospholipids	0.048(±0.028)	0.018-0.111	13
PC(40:4)	glycerophospholipids	0.083(±0.02)	0.05-0.116	11
PC(44:5)	glycerophospholipids	0.162(±0.066)	0.081-0.373	21
PC(40:7)	glycerophospholipids	0.069(±0.006)	0.065-0.072	2
PC(40:8)	glycerophospholipids	0.045(±0.009)	0.036-0.057	5

PC(40:9)	glycerophospholipids	0.028(±0)	0.028-0.028	1
PC(41:5)	glycerophospholipids	0.089(±0.027)	0.06-0.112	3
PC(41:8)	glycerophospholipids	0.031(±0.012)	0.017-0.053	8
PC(42:2)	glycerophospholipids	0.015(±0)	0.015-0.015	1
PC(42:5)	glycerophospholipids	0.067(±0.021)	0.041-0.096	6
PC(42:6)	glycerophospholipids	0.02(±0)	0.02-0.02	1
PC(42:7)	glycerophospholipids	0.072(±0.029)	0.052-0.093	2
PC(42:10)	glycerophospholipids	0.032(±0.006)	0.027-0.036	2
PC(43:2)	glycerophospholipids	0.219(±0)	0.219-0.219	1
PC-O(36:2)	glycerophospholipids	0.186(±0.059)	0.105-0.315	21
PC(44:10)	glycerophospholipids	0.078(±0.041)	0.041-0.136	9
PC(44:12)	glycerophospholipids	0.128(±0.042)	0.101-0.176	3
PC(46:2)	glycerophospholipids	0.16(±0.086)	0.088-0.255	3
PC-O(30:0)	glycerophospholipids	0.085(±0.085)	0.013-0.242	10
PC-O(30:2)	glycerophospholipids	0.059(±0.088)	0.008-0.216	5
PC-O(31:0)	glycerophospholipids	0.161(±0.03)	0.131-0.198	5
AC(4:0-DC)	Acylcarnitines	0.027(±0.016)	0.01-0.068	20
PC-O(32:0)	glycerophospholipids	0.03(±0)	0.03-0.03	1
PC-O(33:1)	glycerophospholipids	0.595(±0.121)	0.384-0.752	12
PC-O(33:3)	glycerophospholipids	0.024(±0.018)	0.011-0.055	5

PC(40:3)	glycerophospholipids	0.053(±0.02)	0.027-0.097	20
PC-O(34:2)	glycerophospholipids	0.043(±0.03)	0.017-0.138	15
PC-O(34:3)	glycerophospholipids	0.012(±0.001)	0.01-0.014	6
PC-O(36:0)	glycerophospholipids	0.07(±0.008)	0.064-0.076	2
PC-O(36:1)	glycerophospholipids	0.117(±0.068)	0.045-0.281	12
PC-O(31:1)	glycerophospholipids	0.415(±0.231)	0.036-0.73	20
PC-O(36:3)	glycerophospholipids	0.321(±0.07)	0.2-0.388	6
PC-O(36:6)	glycerophospholipids	0.021(±0.012)	0.011-0.036	4
PC-O(38:0)	glycerophospholipids	0.112(±0.026)	0.09-0.159	6
PC-O(38:1)	glycerophospholipids	0.219(±0.069)	0.125-0.293	5
PC-O(38:2)	glycerophospholipids	0.102(±0.029)	0.065-0.178	12
PC-O(38:6)	glycerophospholipids	0.051(±0.033)	0.028-0.074	2
PC-O(40:0)	glycerophospholipids	0.043(±0.014)	0.033-0.06	3
PC-O(40:2)	glycerophospholipids	0.12(±0.034)	0.075-0.166	12
PC-O(40:3)	glycerophospholipids	0.122(±0.029)	0.089-0.144	3
PC-O(42:0)	glycerophospholipids	0.058(±0.014)	0.047-0.082	5
PC-O(42:1)	glycerophospholipids	0.078(±0.013)	0.068-0.087	2
PC-O(42:2)	glycerophospholipids	0.059(±0.025)	0.034-0.101	6
PC-O(42:4)	glycerophospholipids	0.052(±0.007)	0.042-0.06	5
PC-O(42:5)	glycerophospholipids	0.086(±0.045)	0.06-0.154	4

PC-O(44:4)	glycerophospholipids	0.085(±0)	0.085-0.085	1
Cer(34:0)	sphingolipids	0.101(±0.058)	0.06-0.167	3
Cer(38:1)	sphingolipids	0.346(±0.244)	0.107-0.635	5
Cer(41:1)	sphingolipids	0.247(±0.034)	0.223-0.271	2
Cer(43:1)	sphingolipids	0.109(±0.039)	0.04-0.129	5
Cer(44:0)	sphingolipids	0.215(±0)	0.215-0.215	1
SM(33:2)	sphingolipids	0.159(±0.149)	0.03-0.437	11
TG(53:4)	glycerides	0.138(±0.039)	0.085-0.248	20
SM(35:1)	sphingolipids	0.204(±0)	0.204-0.204	1
SM(36:1)	sphingolipids	0.154(±0.124)	0.053-0.448	9
SM(36:2)	sphingolipids	0.189(±0.164)	0.024-0.447	5
SM(40:1)	sphingolipids	0.151(±0.088)	0.018-0.261	7
SM(40:4)	sphingolipids	0.062(±0.016)	0.042-0.082	8
SM(41:1)	sphingolipids	0.064(±0)	0.064-0.064	1
SM(42:1)	sphingolipids	0.307(±0)	0.307-0.307	1
SM(42:2)	sphingolipids	0.04(±0)	0.04-0.04	1
SM(42:3)	sphingolipids	0.158(±0.126)	0.057-0.39	7
SM(43:1)	sphingolipids	0.494(±0)	0.494-0.494	1
SM(44:1)	sphingolipids	0.049(±0)	0.049-0.049	1
PC(36:2)	glycerophospholipids	0.248(±0.092)	0.177-0.599	19
CE(16:1)	cholesterol esters	0.977(±0.768)	0.311-1.64	4

CE(17:0)	cholesterol esters	2.82(±0.898)	1.8-3.51	3
PC(40:6)	glycerophospholipids	0.065(±0.024)	0.04-0.129	19
PC-O(33:0)	glycerophospholipids	0.16(±0.081)	0.033-0.288	19
CE(18:3)	cholesterol esters	0.817(±0.54)	0.124-1.3	4
CE(20:4)	cholesterol esters	5.59(±3.46)	2.86-14.4	11
CE(22:6)	cholesterol esters	6.84(±1.53)	5.49-9.78	10
DG(36:3)	glycerides	0.368(±0.069)	0.301-0.465	6
DG(36:4)	glycerides	0.02(±0.006)	0.016-0.024	2
DG(39:0)	glycerides	0.171(±0.11)	0.096-0.359	6
DG(42:0)	glycerides	0.159(±0)	0.159-0.159	1
DG(44:3)	glycerides	0.202(±0.116)	0.062-0.478	15
DG-O(34:1)	glycerides	0.572(±0)	0.572-0.572	1
DG-O(36:4)	glycerides	0.591(±0.025)	0.574-0.609	2
TG(44:2)	glycerides	0.453(±0.095)	0.327-0.611	8
TG(44:4)	glycerides	0.012(±0)	0.012-0.012	1
TG(48:2)	glycerides	0.194(±0.049)	0.11-0.275	19
TG(48:3)	glycerides	0.484(±0)	0.484-0.484	1
TG(49:2)	glycerides	0.24(±0.084)	0.167-0.339	4
TG(50:2)	glycerides	0.155(±0.065)	0.081-0.305	16
TG(50:3)	glycerides	0.224(±0.071)	0.174-0.274	2

TG(50:4)	glycerides	0.016(±0)	0.016-0.016	1
TG(51:1)	glycerides	0.744(±0.079)	0.653-0.791	3
TG(51:2)	glycerides	0.07(±0)	0.07-0.07	1
TG(51:3)	glycerides	0.115(±0.029)	0.094-0.136	2
TG(51:4)	glycerides	0.109(±0.06)	0.036-0.244	15
TG(52:2)	glycerides	0.283(±0.161)	0.116-0.563	8
TG(52:3)	glycerides	0.416(±0.418)	0.145-1.4	8
CE(18:1)	cholesterol esters	1.98(±2.46)	0.21-9.07	18
TG(52:5)	glycerides	0.149(±0)	0.149-0.149	1
TG(52:6)	glycerides	0.251(±0)	0.251-0.251	1
TG(53:3)	glycerides	0.074(±0)	0.074-0.074	1
PC(30:0)	glycerophospholipids	0.063(±0.026)	0.023-0.111	17
TG(53:6)	glycerides	0.153(±0)	0.153-0.153	1
TG(54:3)	glycerides	0.337(±0.003)	0.335-0.339	2
TG(54:4)	glycerides	0.322(±0)	0.322-0.322	1
TG(54:5)	glycerides	0.162(±0.137)	0.078-0.366	4
TG(54:6)	glycerides	0.129(±0.005)	0.126-0.133	2
TG(54:7)	glycerides	0.361(±0)	0.361-0.361	1
TG(55:7)	glycerides	1.54(±1.13)	0.469-2.72	6
TG(55:8)	glycerides	0.107(±0.064)	0.043-0.224	8
TG(56:6)	glycerides	0.136(±0)	0.136-0.136	1

TG(56:7)	glycerides	0.06(±0)	0.06-0.06	1
TG(56:8)	glycerides	0.365(±0.193)	0.235-0.587	3
TG(56:9)	glycerides	0.858(±0.127)	0.768-0.948	2
